# Severe SARS-Cov2 pneumonia in vaccinated patients: a multicenter cohort study

**DOI:** 10.1038/s41598-023-29131-9

**Published:** 2023-02-02

**Authors:** Adrien Mirouse, Alice Friol, Anne-Sophie Moreau, Boris Jung, Edouard Jullien, Côme Bureau, Michel Djibré, Nicolas de Prost, Lara Zafrani, Laurent Argaud, Danielle Reuter, Laure Calvet, Etienne de Montmollin, Sarah Benghanem, Claire Pichereau, Tai Pham, Patrice Cacoub, Lucie Biard, David Saadoun

**Affiliations:** 1 Département de Médecine Interne et Immunologie Cinique, Groupe Hospitalier Pitié-Salpêtrière, APHP, Paris, France; 2 Sorbonne Université, Paris, France; 3 Service de Réanimation Polyvalente, CHU de Lille - Hôpital Roger Salengro, Lille, France; 4 Médecine Intensive et Réanimation, PhyMedExp, Université de Montpellier, Montpellier, France; 5 Service de Médecine Intensive Réanimation, Hôpital Ambroise Paré, APHP, Boulogne, France; 6 Service de Médecine Intensive Réanimation, Département R3S, Hôpital Pitié-Salpêtrière, APHP, Paris, France; 7 Service de Médecine Intensive Réanimation, Université de Paris, Hopital Tenon, APHP, Paris, France; 8 Service de Médecine Intensive Réanimation, Hôpital Henri Mondor, APHP, Créteil, France; 9 Service de Médecine Intensive Réanimation, Hôpital Saint-Louis, APHP, Paris, France; 10 Université de Paris, Paris, France; 11 Service de Médecine Intensive Réanimation, Hôpital Edouard Herriot, Hospices Civils de Lyon, Lyon, France; 12 Service de Réanimation Polyvalente, CH Sud-Francilien, Corbeil-Essonnes, France; 13 Service de Médecine Intensive Réanimation, CHU de Clermont-Ferrand, Clermont-Ferrand, France; 14 Service de Médecine Intensive Réanimation, Hôpital Bichat, APHP, Paris, France; 15 Service de Médecine Intensive Réanimation, Hôpital Cochin, APHP, Paris, France; 16 Service de Médecine Intensive Réanimation, CHI Poissy Saint Germain en Laye, Poissy, France; 17 Service de Médecine Intensive Réanimation, AP-HP, Hôpital de Bicêtre, Hôpitaux Universitaires Paris-Saclay, Le Kremlin-Bicêtre, France; 18 Inserm U1018, Equipe d’Epidémiologie respiratoire intégrative, CESP, Université Paris-Saclay, UVSQ, Univ. Paris-Sud, 94807 Villejuif, France; 19 Service de Biostatistique et Information Médicale, Hôpital Saint-Louis, APHP, Paris, France

**Keywords:** Viral infection, Disease-free survival, Respiratory signs and symptoms, Hypoxia

## Abstract

Vaccination reduces risk of infection, hospitalization, and death due to SARS-Cov2. Vaccinated patients may however experience severe SARS-Cov2 disease. The objective was to describe clinical features of vaccinated patients requiring intensive care unit (ICU) admission due to SARS-Cov2 infection and compare them to a published cohort of unvaccinated patients. We performed a multicenter cohort study of patients with severe SARS-Cov2 disease admitted to 15 ICUs in France between January and September 2021. 100 consecutive vaccinated patients (68 (68%) men, median age 64 [57–71]) were included. Immunosuppression was reported in 38 (38%) patients. Among available serologies at ICU admission, 64% exhibited an optimal antibody level. Median SOFA score at ICU admission was 4 [4–6.3] and median PaO2/FiO2 ratio was 84 [69–128] mmHg. A total of 79 (79%) and 18 (18%) patients received high flow nasal oxygen and non-invasive mechanical ventilation, respectively. Invasive mechanical ventilation (IMV) was initiated in 48 (48%) with a median duration of 11 [5–19] days. During a median ICU length-of-stay of 8 [4–20] days, 31 (31%) patients died. Age (OR per 5-years increment 1.38 CI95% [1.02–1.85], p = 0.035), and SOFA at ICU admission (OR 1.40 CI95% [1.14–1.72] per point, p = 0.002) were independently associated with mortality. When compared to a cohort of 1316 unvaccinated patients (72% men, median age 63 [53–71]), vaccinated patients exhibited less frequently diabetes (16 [16%] vs. 351 [27%], p = 0.029) but were more frequently immunosuppressed (38 [38%] vs. 109 (8.3%), p < 0.0001), had more frequently chronic kidney disease (24 [24%] vs. 89 (6.8%), p < 0.0001), chronic heart failure (16 [16%] vs. 58 [4.4%], p < 0.0001), and chronic liver disease (3 [3%] vs. 8 [0.6%], p = 0.037) compared to unvaccinated patients. Despite similar severity, vaccinated patients required less frequently IMV at ICU day 1 and during ICU stay (23 [23%] vs. 785 [59.7%], p < 0.0001, and 48 [48%] vs. 930 [70.7%], p < 0.0001, respectively). There was no difference concerning ICU mortality (31 [31%] vs. 379 [28.8%], p = 0.64). Severe SARS-Cov2 infection after vaccination occurs mainly in patients with immunosuppression, chronic kidney, heart or liver failure. Age and disease severity are independently associated with mortality.

## Introduction

Severe Acute Respiratory Syndrome-Coronavirus 2 (SARS-Cov2) pandemic is one of the most challenging health situation that has developed over the past decades, with more than 300 million confirmed cases and more than 5 million deaths at the beginning of year 2022^1^. A cornerstone of pandemic control is the widespread administration of vaccines. Vaccination objectives are to prevent infection and spreading but also to prevent severe infection requiring hospitalization and intensive care unit (ICU) admission.

In Western countries, vaccination programs started between December 2020 and January 2021. The main achievement in the face of this global challenge has been the decrease in the number of hospitalizations^2^. However, there are increasing reports of breakthrough infection among persons who have been vaccinated against the SARS-Cov2^3–6^. Most of these reports are population-based and do not give the clinical picture of vaccinated patients who develop severe disease requiring ICU admission. Given the evolution of the pandemic and widespread vaccination, data are needed to identify patients at higher risk of severe infection despite repeated vaccination.

Our objective was to report the clinical picture, management and outcomes of patients vaccinated against SARS-Cov2 and who developed a severe SARS-Cov2 infection requiring ICU admission. The secondary objective was to compare those patients with unvaccinated SARS-Cov2 critically ill patients to help to guide public policies in higher risk patients.

## Results

### Study population

The first 100 consecutive vaccinated patients admitted for the management of SARS-Cov2 pneumonia in the participating ICU were included between January and September 2021, including 68 (68%) men and with a median age of 64 [57–71] years (Table 1). Main comorbidities and severe SARS-Cov2 infection risk factors were hypertension in 47 (47%) patients, obesity in 40 (40%) patients, immunosuppression in 38 (38%) patients, dyslipidemia in 23 (24%) patients, chronic kidney disease in 24 (24%) patients, diabetes in 16 (16%) patients, and chronic heart failure in 16 (16%) patients. Main immunosuppression causes were solid organ transplantation in 17 (17%) patients, including kidney transplantation in 12 patients, liver transplantation in 3 patients and heart transplantation in 2 patients; solid cancer in 11 (11%) patients; hematological malignancies in 9 (9%) patients; auto-immune diseases in 3 (3%) patients, including one rheumatoid arthritis, one systemic sclerosis, one myasthenia gravis; and one Bruton agammaglobulinemia. No patient had a previously documented SARS-Cov2 infection before the ICU associated episode.

**Table 1:** Characteristics of 100 vaccinated patients with critically ill SARS-Cov2 infection.

Characteristics	All patients n = 100
Gender, man	68 (68%)
Age, years	64 [57–71]
Body mass index, kg/m^2^	29.2 [24.7–32.6]
Cardiovascular risk factors	58 (59%)
Hypertension	47 (47%)
Dyslipidemia	23 (24%)
Diabetes	16 (16%)
Chronic heart failure	16 (16%)
Chronic respiratory disease	9 (9%)
Chronic kidney disease	24 (24%)
Immunosuppression	38 (38%)
Solid organ transplantation	17 (17%)
Chronic steroids	16 (16%)
Solid cancer	11 (11%)
Hematological malignancy	9 (9%)
Charlson comorbidity index	4 [2.5–6]
Frailty score	2 [2–3]
Day 1 SOFA	4 [4–6.25]
Temperature	37.5 [36.9–38.0]
Blood analysis at ICU day 1	
Leukocytes, G/L	8.2 [5.9–12.2]
Hemoglobin, g/dL	12.5 [11.2–13.7]
Platelets, G/L	219 [151–299]
Creatinin, µmol/L	92 [68–174]
CRP, mg/L	116 [48–181]
PaO_2_/FiO_2_	84 [69–128]
Lactate, mmol/L	1.3 [1–1.9]
LDH, UI/L	460 [355–605]
D-dimers, UI/L	1367 [790–3366]
Chest computed tomography-scan	82 (89%)
SARS-Cov2 pneumonia lesions extension, %	60 [50–75]
Pulmonary embolism, yes, n (%)	6 (10%)
Positive SARS-Cov2 serology	9 (64%)

*CRP* C-reactive protein, *ICU* intensive care unit, *LDH* lactate dehydrogenase, *SARS-Cov2* severe acute respiratory syndrome coronavirus 2, *SOFA* sequential organ failure assessment.

### Vaccination

Among all patients, 80 (80%) had received at least one injection, 18 (18%) two injections, and 2 (2%) three injections. Among the 80 patients with one injection, 73 (91%) presented the first SARS-Cov2 infection symptoms at least 14 days after the injection. Vaccines were distributed as following: comirnaty in 74 (74%) patients, ChAdOx1-S in 20 (20%) patients, mRNA-1273 in 4 (4%) patients, and Ad26COV2.S in 2 (2%) patients. Serological status for SARS-Cov2 at ICU admission was available in 14 (14%) patients and, among them, 9 (64%) exhibited a positive SARS-Cov2 serology.

### ICU admission and management

Among all patients, 98 (98%) were admitted for the management of acute respiratory failure and 2 (2%) for the management of cardiac arrest. Median SOFA and SAPS II scores at ICU admission were 4 [4–6.25], and 32 [27–42], respectively. Respiratory supports at ICU admission and during ICU stay are displayed in Table 2. All patients received standard oxygen therapy. Median PaO_2_/FiO_2_ ratio at ICU admission was 84 [69–128] mmHg. High flow nasal oxygen and non-invasive mechanical ventilation were implemented in 79 (79%) and 18 (18%) patients, respectively. Mechanical ventilation was initiated in 48 (48%) with a median duration of 11 [5–19] days. Acute kidney injury was diagnosed in 25 (25%) patients and 11 (11%) required renal replacement therapy. At ICU admission, 13 (13%) patients required vasopressors and 18 (18%) other patients developed a shock during ICU management.

**Table 2:** ICU management and outcomes.

Characteristics	Patients n = 100
Oxygenation	
Standard oxygen therapy	100 (100%)
Duration, days	1 [0–3]
High flow nasal oxygen	79 (79%)
Duration, days	3 [2–5]
Non-invasive ventilation	18 (18%)
Duration, days	2.5 [1.8–4]
Invasive mechanical ventilation	48 (48%)
Duration, days	11 [5–19]
Prone position	35 (36%)
Number	3.0 [2.0–5.3]
ECMO	3 (3%)
Duration, days	11 [10–18]
Organ supply	
Renal replacement therapy	11 (11%)
Vasopressors at day 1	13 (13%)
Vasopressors	31 (31%)
SARS-Cov2 pneumonia treatment	
Dexamethasone	88 (89%)
Duration, days	10 [10–10]
Tocilizumab	30 (30%)
Convalescent patient plasma therapy	9 (9%)
Monoclonal antibodies	4 (4%)
Outcomes	
Superinfection	34 (34%)
ICU length-of-stay, days	8 [4–20]
ICU length-of-stay in survivors, days	10 [3.5–21]
Hospital length-of-stay, days	21 [11–32]
Mortality	31 (31%)

*ECMO* extra-corporal membrane oxygenation, *ICU* intensive care unit, *SARS-Cov2* severe acute respiratory syndrome coronavirus 2.

For the SARS-Cov2 pneumonia specific treatment, 88 (89%) patients received dexamethasone during 10 [10] days, and 30 (30%) patients received tocilizumab. Convalescent patients’ plasma and specific monoclonal antibodies were administered to 9 (9%) and 4 (4%) patients, respectively.

### Outcomes

**Figure 1:**
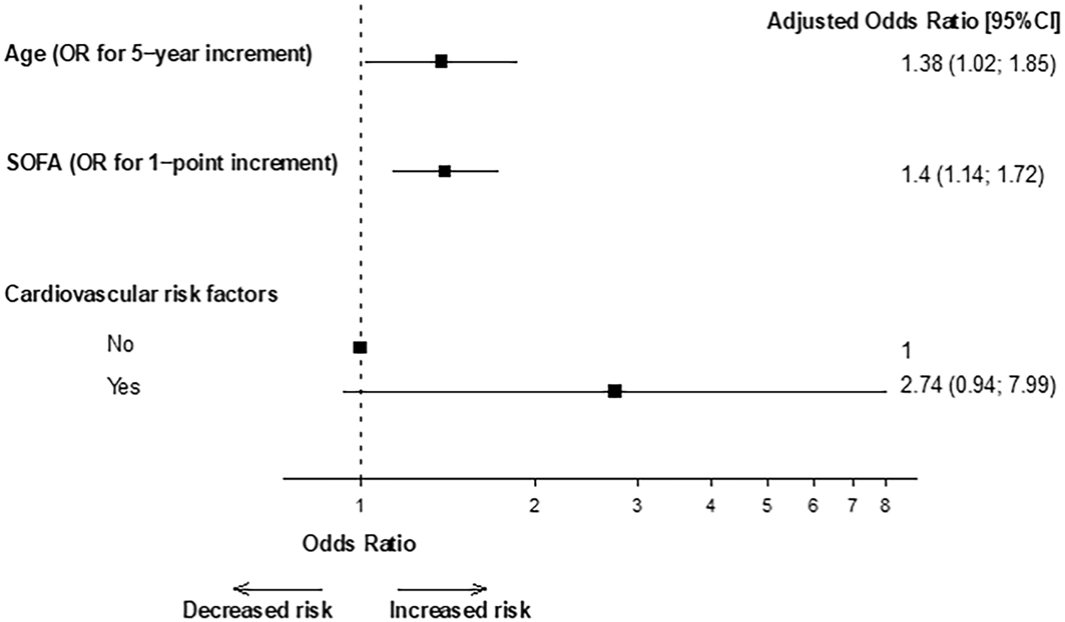
Multivariate analysis of factors associated with mortality.

During a median ICU length-of-stay of 8 [4–20] days, 34 (34%) patients exhibited a respiratory superinfection, and 31 (31%) patients died (Table 2). Main causes of death were refractory ARDS in 16 (52%) patients, multi-organ failure in 11 (35%) patients, limitation of life support because of an expected poor outcome in 3 (10%) patients, and septic shock secondary to ventilator-acquired pneumonia in one (3%) patient. In univariate analysis, age (OR 1.39 CI95% [1.08–1.78] per 5-years increment, p = 0.011), cardiovascular risk factors (OR 3.68 CI95% [1.40–9.66], p = 0.008), and SOFA at ICU admission (OR 1.39 CI95% [1.14–1.68] per point, p = 0.001) were associated with mortality (Table 3). In multivariate analysis, age (OR 1.38 CI95% [1.02–1.85] per 5-years increment, p = 0.035), and SOFA at ICU admission (OR 1.40 CI95% [1.14–1.72] per point, p = 0.002) were independently associated with mortality (Fig. 1).

**Table 3:** Factors associated with mortality.

Variables	Univariates analysis	p-value	Multivariate analysis	p-value
Gender, men	1.64 [0.64–4.20]	0.31		
Age, per 5 years	1.39 [1.08–1.78]	0.011	1.38 [1.02–1.85]	0.035
Cardiovascular risk factors	3.68 [1.40–9.66]	0.008	2.74 [0.94–7.99]	0.066
Hypertension	3.01 [1.25–7.25]	0.014		
Dyslipidemia	2.47 [0.94–6.50]	0.066		
Diabetes	3.35 [1.12–10.1]	0.031		
Chronic heart failure	1.32 [0.43–4.00]	0.63		
Chronic pulmonary disease	1.45 [0.38–5.56]	0.59		
Immunosuppression	1.73 [0.73–4.07]	0.21		
Cancer*	1			
Systemic diseases	1.47 [0.18–11.7]	0.72		
Solid organ transplantation	1.96 [0.47–8.11]	0.36		
Charlson comorbidity index	1.17 [0.97–1.40]	0.096		
Vaccination				
Time between first injection and ICU admission > 14 days	1.16 [0.45–3.04]	0.76		
No. of injections				
One injection	1			
Two injections	0.37 [0.10–1.39]	0.14		
Three injections	1.86 [0.11–30.8]	0.67		
SOFA day 1	1.39 [1.14–1.68]	0.001	1.40 [1.14–1.72]	0.002

*SOFA* sequential organ failure assessment.

*Solid cancer and hematological malignancies.

### Comparison with unvaccinated patients

Comparison with a published cohort of SARS-Cov2 critically-ill unvaccinated patients is displayed in Table 4. Patients were comparable for demographic data with similar age, gender, and BMI. Vaccinated patients exhibited less frequently diabetes (16 [16%] vs. 351 [27%], p = 0.019) but more frequently immunosuppression (38 [38%] vs. 109 (8.3%), p < 0.0001), chronic kidney disease (24 [24%] vs. 89 (6.8%), p < 0.0001), chronic heart failure (16 [16%] vs. 58 [4.4%], p < 0.0001), and chronic liver disease (3 [3%] vs. 8 [0.6%], p = 0.037). Severity, assessed by SOFA at ICU admission, was similar between the two groups (4 [4–6] vs. 4 [3–6], p > 0.99). Vaccinated patients had a significantly lower PaO_2_/FiO_2_ ratio at ICU admission (84 [69–128] vs. 125 [90–180], p < 0.0001). Despite similar severity, vaccinated patients required less frequently IMV at ICU day 1 and during ICU stay (23 [23%] vs. 785 [59.7%], p < 0.0001, and 48 [48%] vs. 930 [70.7%], p < 0.0001, respectively). There was no difference concerning ICU mortality (31 [31%] vs. 379 [28.8%], p = 0.72).

**Table 4:** Comparison between unvaccinated and vaccinated critically-ill patients.

Characteristics	Unvaccinated patients^32^ n = 1316	Vaccinated patients n = 100	p-value
Age, years	63 (53–71)	64 (57–71)	0.35
Gender, men	942 (71.6%)	68 (68%)	0.45
BMI, kg/m^2^	29 (26–32)	29 (25–33)	0.8
Comorbidities			
Hypertension	650 (49.4%)	47 (48%)	0.71
Obesity	557 (42.3%)	40 (47%)	0.45
Diabetes mellitus	351 (26.7%)	16 (16%)	0.019
Immunosuppression	109 (8.3%)	38 (38%)	< 0.0001
Chronic kidney disease	89 (6.8%)	24 (24%)	< 0.0001
Chronic heart failure	58 (4.4%)	16 (16%)	< 0.0001
Hematological disease	35 (2.7%)	9 (9%)	0.003
Chronic liver disease	8 (0.6%)	3 (3%)	0.037
Severity of illness			
SOFA	4 (3–6)	4 (4–6)	> 0.99
PaO_2_/FiO_2_, mmHg	125 (90–180)	84 (69–128)	< 0.0001
ARDS	1268 (97%)	87 (87%)	0.0002
Respiratory support at admission			
Standard oxygen therapy	158 (12%)	86 (86%)	< 0.0001
HFNO	641 (48.7%)	77 (77%)	< 0.0001
NIV	89 (6.8%)	14 (14%)	0.007
IMV	785 (59.7%)	23 (23%)	< 0.0001
Respiratory support during ICU stay			
IMV	930 (70.7%)	48 (48%)	< 0.0001
Prone	721 (54.8%)	135 (36%)	0.0004
ECMO	30 (2.3%)	3 (3%)	0.49
Pneumonia	328 (24.9%)	31 (31%)	0.18
Organ failure			
AKI	298 (22.6%)	25 (25%)	0.59
Shock at admission	306 (23.3%)	13 (13%)	0.018
Treatments			
Steroids	1262 (95.9%)	88 (88%)	0.004
Tocilizumab	82 (6.2%)	30 (30%)	< 0.0001
Outcomes			
ICU length-of-stay, days			
Survivors	12 (7–27)	10 (3.5–10.5)	–
Non survivors	19 (12–29)	14 (4–16)	–
ICU mortality	379 (28.8%)	31 (31%)	0.72

*AKI* acute kidney injury, *ARDS* acute respiratory distress syndrome, *ECMO* extra-corporal membrane oxygenation, *HFNO* high-flow nasal oxygen, *ICU* intensive care unit, *IMV* invasive mechanical ventilation, *NIV* non-invasive ventilation, *SOFA* sequential organ failure assessment.

## Discussion

In this multicenter cohort study, we retrospectively identified vaccinated critically-ill SARS-Cov2 patients with data prospectively collected in each center. We sought to describe their clinical features, management, identify factors associated with mortality, and to compare these patients with critically-ill unvaccinated patients. We found that nearly 40% of vaccinated critically-ill SARS-Cov2 patients had underlying immunosuppression. Nearly half of the patients required mechanical ventilation. Age and severity at ICU admission were independently associated with mortality. Despite similar demographics and severity at ICU admission and lower PaO_2_/FiO_2_ ratio, vaccinated patients required less often mechanical ventilation as compared to critically-ill SARS-Cov2 unvaccinated patients.

Recent large cohorts have described the occurrence of SARS-Cov2 infection in vaccinated patients. In a Scottish prospective study, it was reported that < 0.1% of the population was admitted to the hospital or died due to SARS-Cov2 infection^3^. They identified older age (≥ 80 years), comorbidities, recent hospitalization, socioeconomic disadvantage, and being an ex-smoker as risk factors for post-vaccination SARS-Cov2 infection. In this cohort, critically-ill patients were not described. Another large cohort population compared the risk of developing severe/critical disease between vaccinated and unvaccinated veterans^6^. Vaccination was protective against critical disease (OR 0.55 CI95% [0.45–0.68]) whereas age and comorbidities accumulation (OR 1.25 CI95% [1.11–1.41] per 10-years and OR 2.85 CI 95% [1.49–5.43], respectively) were poor prognosis factors. ICU management and clinical picture of critically-ill patients were not provided. An English national cohort reported various conditions, including Down’s syndrome, kidney transplantation, sickle cell disease, care home residency, chemotherapy, AIDS, liver cirrhosis, neurological conditions, transplantation, chronic kidney disease, and cardiovascular risk factors as associated with mortality due to SARS-Cov2 infection in vaccinated patients^7^. However, this cohort included elderly patients with most deaths (78%) occurring after 80 years-old. Most of these elderly and comorbid patients could have not been eligible to ICU admission and information provided by these cohorts differ from our results.

In our cohort, age and disease severity were associated with mortality. Comorbidities were not associated with mortality, as previously reported^3,7,8^. However, these findings were reported in general population. As we report patients admitted to ICU, patients with more comorbidities might have been denied from ICU admission because of an expected poor outcome. One other study and one other letter have pointed out that critically-ill vaccinated patients were older, more comorbid, without a higher ICU-mortality^9,10^.

Vaccination has proven effective in preventing symptomatic SARS-Cov2 infection, hospitalization, and severe disease. Despite these elements, few vaccinated patients continue to develop severe disease requiring ICU admission. In our study, when compared to unvaccinated critically-ill patients and despite similar severity at ICU admission, vaccinated patients required less often mechanical ventilation but had a similar death ratio. We could hypothesize that vaccination could inflect the disease course in ICU-admitted patients.

There is a need to better understand these patients’ condition and to identify risk factors of developing severe SARS-Cov2 infection after vaccination. Serological response has been proposed as a surrogate marker of disease risk after vaccination with various cut-off limits^11–14^. However, more recent studies revealed the imperfect correlation between post-vaccination serological response and disease risk^15^. Neutralizing antibodies titer may decrease over time but does not necessary reflect a higher risk of infection as anamnestic antibody response may occur lately in case of re-exposition^16–18^. A correlate of protection for SARS-Cov2 vaccines is urgently needed^19^. In our study, 64% of patients with a serological status at ICU admission had a positive serology after vaccination and before the occurence of severe infection. These results are in line with another cohort evaluating the antibodies titer at ICU admission in vaccinated patients^4^. Altered antibody generation might reflect a defective germinal center response in some immunocompromised patients with altered cellular response^20^. Cellular response assessment based on interferon-gamma release assays has been proposed to identify a correct response in patients with deficient humoral response^21,22^. In our study, we identified patients with immunosuppression or chronic kidney, heart or liver failure as at higher risk to required ICU admission for the management of post-vaccination SARS-Cov2 infection. Solid organ transplantation, solid cancer, and hematological malignancies outnumbered the other causes of immunosuppression, including systemic diseases. These results are in line with other studies pointing out that, despite the use of similar immunosuppressive drugs, patients with rheumatic and musculoskeletal diseases generally do not exhibit a higher risk of SARS-Cov2 infection even in patients with low antibody response^23^. Recent studies reported that SARS-Cov2 vaccination induces immunological T-cell memory able to cross-recognize SARS-Cov2 variants from Alpha to Omicron, despite the Omicron variant escaping neutralizing antibodies^24–26^.

Our study has several strengths. It is one the first study to precisely describe the clinical picture of vaccinated critically-ill SARS-Cov2 patients. It might help to identify patients requiring extra-vaccination preventive measures. It confirms the benefit of vaccination, even in most severe patients. We also acknowledge several limits. Vaccinated patients were identified retrospectively. However, data on critically-ill SARS-Cov2 patients are prospectively collected in each participating center. It suggests that very few vaccinated patients may have been missed for inclusion. We compared our cohort with an already published Spanish national cohort. This cohort was interesting because including only ICU-admitted patients. We chose to exclude first waves patients and to compare with the second/third wave of patients because resuscitation practices were more similar with our practices over the same period in 2021. This way, we believe our results are more applicable. However, differences in standard of care between countries and period may bias some of the results, including the use of non-invasive ventilation devices.

## Conclusion

Severe SARS-Cov2 infection occurs post-vaccination essentially in patients with immunosuppression, chronic kidney, heart or liver failure. Age and disease severity are independently associated with mortality. Vaccination might inflect the disease course, even in critically-ill patients.

## Methods

This study was approved by the French Intensive Care Society Ethics Committee (#21–51) on May the 17th 2021, and received an authorization to use patients’ data from the French Data Protection Agency. According to French law, a waiver of informed consent was obtained (French Intensive Care Society Ethics Committee (#21–51)). All methods were performed in accordance with the relevant guidelines and regulations.

### Study design, setting, and population

We conducted a multicenter retrospective cohort study in 15 French ICUs (see Supplementary Appendix: e-Table 1). We identified all adults (≥ 18 years of age) admitted to the participating ICUs between January 1, 2021, and September 1, 2021, and infected with SARS-Cov2. Sample size was defined by the number of consecutive patients with inclusion criteria, admitted to ICU during the study period. All patients’ medical records were reviewed by the local investigator to check the SARS-Cov2 vaccine status. Each selected records were reviewed by 2 investigators (AM and AF) to confirm the inclusions criteria. The diagnosis of SARS-Cov2 pneumonia was made if the patient presented respiratory symptoms and a SARS-Cov2 positive PCR in nasal swab or respiratory samples. Vaccination status was defined according to patients and/or patients’ relatives declaration. All patients with at least one SARS-Cov2 vaccine injection were included. Patients were considered efficiently vaccinated if there was a 14-day minimum delay between first vaccine injection and first SARS-Cov2 infection symptoms. This definition was used in population-wide cohorts^3,7^.

### Data collection

For each patient, data were extracted manually from the medical chart. We obtained data for baseline patient characteristics, including demographics, comorbidities, chronic medications, and past medical history. Clinical and laboratory data, and imaging were extracted from the medical charts. High risk factors for severe SARS-Cov2 infection were retrieved, including immunosuppression, chronic kidney disease, diabetes, hypertension, and obesity. Obesity was defined as a body mass index (BMI) ≥ 30 kg/m^2^. Immunosuppression was defined as chronic use of steroids (> 3 months of more or equal to 7.5 mg/day of prednisone) or other immunosuppressive drugs, solid organ transplantation, HIV infection, hematologic malignancy or solid tumor active or treated by chemotherapy during the past 5 years. Disease severity was assessed using the Sequential Organ Failure Assessment (SOFA) and simplified Acute Physiology Score (SAPS II) at day 1 after ICU admission^27,28^. Patients were classified as having acute respiratory distress syndrome (ARDS) according to the Berlin definition^29^. Hypoxemia severity was assessed using the PaO_2_/fraction of inspired oxygen (FiO_2_) ratio^30^. Acute kidney injury was defined according to the Kidney Disease Improving Global Outcomes criteria^31^. We extracted the use of life-sustaining treatments (i.e., standard oxygen therapy, noninvasive or invasive mechanical ventilation, high flow nasal oxygen [HFNO], renal replacement therapy, and vasopressors) from medical chart. Therapeutic regimens were reported, including antiviral therapy (molecule, dose, and length of treatment), the use of steroids, tocilizumab, convalescent plasma treatment. Two physicians (AM and AF) assessed outcomes by reviewing the medical charts or contacting the local investigator, concerning ICU and hospital mortality.

### Comparison with unvaccinated critically-ill SARS-Cov2 patients

In order to compare presentation and outcomes between vaccinated and unvaccinated critically-ill SARS-Cov2 patients, our cohort was compared to a large European cohort of wave 2/3 critically ill SARS-Cov2 patients^32^. Data were extracted from the publication and compared to our cohort to identify differences in demographics, presentation, and outcomes of vaccinated and unvaccinated patients.

### Statistical analysis

Categorical variables are summarized as counts (percentages), and continuous variables are summarized as medians (interquartile ranges, IQR). Factors associated with hospital mortality were examined in univariable analysis using logistic regression models. An adjusted multivariable model was selected using a stepwise procedure based on Akaiké’s information criterion. For continuous variable, the log-linearity assumption was checked by comparing linear modeling with models using restricted cubic splines. Results of this in-hospital mortality analysis are reported with odds ratios and their 95% confidence intervals. For the comparison of our cohort with Carbonell et al.’s 2/3 wave cohort^32^, count data were retrieved from their results tables and straightforwardly compared using standard χ^2^ test, or Fisher’s exact test whenever χ^2^ test was not valid. For quantitative variables, given that Carbonell et al.’s reported them as medians (IQR), and that we did not have access to individual data from their cohort, we used z-tests assuming roughly normal-distributed variables (medians roughly equal to means, computing estimated standard deviations from the IQR boundaries). Detailed calculations are available in supplementary material (e-Table 2). All tests were two-sided at the 5%-level. Analyses were performed using R statistical platform, version 4.0.1.

## Supplementary Information

Supplementary Tables.

## Data Availability

The datasets used and/or analyzed during the current study are available from the corresponding author on reasonable request.

## Abbreviations

AKI: Acute kidney injury
ARDS: Acute respiratory distress syndrome
BMI: Body mass index
CI95%: Confidence interval 95%
FiO_2_: Fraction of inspired oxygen
HFNO: High-flow nasal oxygen
ICU: Intensive care unit
IQR: Interquartile ranges
IMV: Invasive mechanical ventilation
NIV: Non-invasive mechanical ventilation
OR: Odd-ratio
SARS-Cov2: Severe acute respiratory syndrome coronavirus 2
SOFA: Sequential organ failure assessment

## References

[1] *WHO Coronavirus (COVID-19) Dashboard*. https://covid19.who.int.

[2] León TM (2022). COVID-19 cases and hospitalizations by COVID-19 vaccination status and previous COVID-19 diagnosis—California and New York, May-November 2021. Morb. Mortal. Wkly. Rep..

[3] Agrawal U (2021). COVID-19 hospital admissions and deaths after BNT162b2 and ChAdOx1 nCoV-19 vaccinations in 2.57 million people in Scotland (EAVE II): A prospective cohort study. Lancet Respir. Med..

[4] Estella Á (2021). Vaccinated patients admitted in ICU with severe pneumonia due to SARS-CoV-2: A multicenter pilot study. J. Pers. Med..

[5] Cook C (2022). Clinical characteristics and outcomes of COVID-19 breakthrough infections among vaccinated patients with systemic autoimmune rheumatic diseases. Ann. Rheum. Dis..

[6] Butt AA, Yan P, Shaikh OS, Mayr FB, Omer SB (2021). Rate and risk factors for severe/critical disease among fully vaccinated persons with breakthrough SARS-CoV-2 infection in a high-risk national population. Clin. Infect. Dis..

[7] Hippisley-Cox J (2021). Risk prediction of covid-19 related death and hospital admission in adults after covid-19 vaccination: National prospective cohort study. BMJ..

[8] Ioannou GN (2021). COVID-19 vaccination effectiveness against infection or death in a national US health care system: A target trial emulation study. Ann. Intern. Med..

[9] Otto M (2022). Clinical characteristics and outcomes of critically ill patients with one, two and three doses of vaccination against COVID-19 in Australia. Intern. Med. J..

[10] Hilty MP (2022). mRNA-based SARS-CoV-2 vaccination is associated with reduced ICU admission rate and disease severity in critically ill COVID-19 patients treated in Switzerland. Intens. Care Med..

[11] Gilbert PB (2021). Immune correlates analysis of the mRNA-1273 COVID-19 vaccine efficacy clinical trial. Science..

[12] Levin EG (2021). Waning immune humoral response to BNT162b2 covid-19 vaccine over 6 months. N. Engl. J. Med..

[13] Khoury DS (2021). Neutralizing antibody levels are highly predictive of immune protection from symptomatic SARS-CoV-2 infection. Nat. Med..

[14] Zhu F (2022). WHO international standard for SARS-CoV-2 antibodies to determine markers of protection. Lancet Microbe.

[15] Rydyznski Moderbacher C (2020). Antigen-specific adaptive immunity to SARS-CoV-2 in acute COVID-19 and associations with age and disease severity. Cell.

[16] Gagne M (2022). Protection from SARS-CoV-2 delta one year after mRNA-1273 vaccination in rhesus macaques coincides with anamnestic antibody response in the lung. Cell.

[17] Sokal A (2021). Maturation and persistence of the anti-SARS-CoV-2 memory B cell response. Cell.

[18] Goel RR (2021). mRNA vaccines induce durable immune memory to SARS-CoV-2 and variants of concern. Science..

[19] Krammer F (2021). A correlate of protection for SARS-CoV-2 vaccines is urgently needed. Nat. Med..

[20] Lederer K (2022). Germinal center responses to SARS-CoV-2 mRNA vaccines in healthy and immunocompromised individuals. Cell..

[21] Prendecki M (2021). Humoral and T-cell responses to SARS-CoV-2 vaccination in patients receiving immunosuppression. Ann. Rheum. Dis..

[22] Mudd PA (2021). SARS-CoV-2 mRNA vaccination elicits a robust and persistent T follicular helper cell response in humans. Cell..

[23] Kroon FPB (2021). Risk and prognosis of SARS-CoV-2 infection and vaccination against SARS-CoV-2 in rheumatic and musculoskeletal diseases: A systematic literature review to inform EULAR recommendations. Ann. Rheum. Dis..

[24] Tarke A (2022). SARS-CoV-2 vaccination induces immunological T cell memory able to cross-recognize variants from alpha to omicron. Cell..

[25] Keeton R (2022). T cell responses to SARS-CoV-2 spike cross-recognize omicron. Nature..

[26] GeurtsvanKessel CH (2022). Divergent SARS CoV-2 omicron-reactive T- and B cell responses in COVID-19 vaccine recipients. Sci. Immunol..

[27] Le Gall JR, Lemeshow S, Saulnier F (1993). A new simplified acute physiology score (SAPS II) based on a European/North American multicenter study. JAMA.

[28] Vincent JL (1996). The SOFA (Sepsis-related Organ Failure Assessment) score to describe organ dysfunction/failure. On behalf of the Working Group on Sepsis-Related Problems of the European Society of Intensive Care Medicine. Intens. Care Med..

[29] ARDS Definition Task Force (2012). Acute respiratory distress syndrome: The Berlin definition. JAMA.

[30] Vincent J-L (2009). International study of the prevalence and outcomes of infection in intensive care units. JAMA.

[31] *Acute Kidney Injury (AKI)—KDIGO*. http://kdigo.org/guidelines/acute-kidney-injury/.

[32] Carbonell R (2021). Mortality comparison between the first and second/third waves among 3,795 critical COVID-19 patients with pneumonia admitted to the ICU: A multicentre retrospective cohort study. Lancet Reg. Health Eur..

