# Supplementary material for: Severe SARS-Cov2 pneumonia in vaccinated patients: a multicenter cohort study
Source: Sci Rep. 2023 Feb 2;13:1902. doi: 10.1038/s41598-023-29131-9 (PMC9893202; doi:10.1038/s41598-023-29131-9)
Supplement: Supplementary file 1 — Supplementary Tables. [file 41598_2023_29131_MOESM1_ESM.docx]

**Supplementary data:**

**e-Table 1: participating centers:**

| **Center** | **Number of patients** |
| --- | --- |
| CHRU de Lille | 16 |
| CHU de Montpellier | 12 |
| Hôpital Ambroise Paré, Boulogne | 10 |
| Hôpital Pitié-Salpêtrière, Paris | 9 |
| Hôpital Tenon, Paris | 7 |
| Hôpital Henri Mondor, Créteil | 6 |
| Hôpital Saint-Louis, Paris | 6 |
| Hôpital Edouard Herriot, Lyon | 6 |
| CHU de Clermont-Ferrand | 5 |
| CH Sud-Francilien, Corbeil-Essonnes | 5 |
| Hôpital Bichat, Paris | 4 |
| Institut Gustave Roussy, Villejuif | 4 |
| Hôpital Cochin, Paris | 3 |
| CH de Poissy Saint-Germain-en-Laye, Poissy | 3 |
| Hôpital de Bicêtre, Kremlin Bicêtre | 2 |

**e-Table 2**

**Comparison to unvaccinated critically-ill SARS-Cov2 patients using Carbonell et al’s 2/3 wave cohort of n=1316 patients [1].**

Count data were retrieved from their results tables and straightforwardly compared using standard χ² test, or Fisher’s exact test whenever χ² test was not valid. For quantitative variables, given that Carbonell et al’s reported them as medians (IQR), and that we did not have access to individual data from their cohort, we used z-tests assuming roughly normal-distributed variables (medians roughly equal to means and back-computing estimated standard deviations from the reported IQR boundaries). Details for each variable are provided in the table below.

**Comparison to unvaccinated critically-ill SARS-Cov2 patients using Carbonell et al’s 2/3 wave cohort of n=1316 patients [1].** Unless otherwise specified, compared samples included 1316 patients for the external unvaccinated cohort, and 100 patients for our cohort.

| **Characteristics** | **Patients**  **N=1316** ^12^ | **Patients**  **N=100** | **P – value** |
| --- | --- | --- | --- |
| Age | 63 (53-71)  sd=13.433 | 64 (57 – 71)  sd=10.159 | 0.35 |
| Gender (male) | 942 (71.6%) | 68 (68%) | 0.45 |
| BMI | 29 (26-32)  sd=4.478 | 29 (25 – 33)  sd=8.329, n=86 | 0.8 |
| **Comorbidities** | | | |
| Hypertension | 650 (49.4%) | 47 (47.5%)  N=99 | 0.71 |
| Obesity | 557 (42.3%) | 40 (46.5%)  N=96 | 0.45 |
| Diabetes mellitus | 351 (26.7%) | 16 (16%)  N=99 | 0.019 |
| Immunosuppression | 109 (8.3%) | 38 (38%) | < 0.0001 |
| Chronic kidney disease | 89 (6.8%) | 24 (24.5%)  *n=96* | < 0.0001 |
| Chronic heart failure | 58 (4.4%) | 16 (16%)  *n=99* | < 0.0001 |
| Hematological disease | 35 (2.7%) | 9 (9%)  *n=99* | 0.003* |
| Chronic liver disease | 8 (0.6%) | 3 (3%) | 0.037 |
| **Severity of illness** | | | |
| SOFA | 4 (3-6)  *sd=2.239* | 4 (4-6.25)  *sd=2.568* | >0.99 |
| PaO_2_/FiO_2_ | 125 (90-180)  *sd=93.28* | 84 (69–128)  *sd=86.71* | < 0.0001 |
| ARDS | 1268 (96.4%) | 87 (87%) | 0.0002* |
| **Respiratory support at admission** | | | |
| O2 | 158 (12%) | 86 (86%) | < 0.0001 |
| HFNO | 641 (49%) | 77 (77%) | < 0.0001 |
| NIV | 89 (6.8%) | 14 (14%) | 0.007 |
| IMV | 785 (59.7%) | 23 (23%) | < 0.0001 |
| Shock at admission | 306 (23.3%) | 13 (42%) | 0.018 |
| AKI | 298 (22.6%) | 25 (25%) | 0.59 |
| **Respiratory support during ICU stay** | | | |
| IMV | 930 (70.7%) | 48 (48.5%)  *n=99* | < 0.0001 |
| Prone | 721 (55%) | 35 (36%)  *n=97* | 0.0004 |
| ECMO | 30 (2.3%) | 3 (3%)  *n=97* | 0.49* |
| **Organ failure** | | | |
| Pneumonia | 328 (25%) | 31 (31%)  *n=99* | 0.18 |
| **Treatments** | | | |
| Steroids | 1262 (96%) | 88 (88%)  *n=99* | 0.004* |
| Tocilizumab | 82 (6.2%) | 30 (30%) | < 0.0001 |
| **Outcomes** | | | |
| ICU LOS, days |  |  |  |
| Survivors | 12 (7-27) | 10 (3.5 – 10.5) | - |
| Non survivors | 19 (12-29) | 14 (4 – 16) | - |
| ICU Mortality | 379 (29%) | 31 (31%) | 0.72 |

* p-value from Fisher’s exact test (Pearson’s χ² not applicable).

Abbreviations : sd=estimated standard deviation used for the z-test ; N=sample size

[1] Carbonell R, Urgelés S, Rodríguez A, Bodí M, Martín-Loeches I, Solé-Violán J, et al. Mortality comparison between the first and second/third waves among 3,795 critical COVID-19 patients with pneumonia admitted to the ICU: A multicentre retrospective cohort study. Lancet Reg Health – Eur. 2021;11. Available at: <https://www.thelancet.com/journals/lanepe/article/PIIS2666-7762(21)00229-5/fulltext>
